# The impact of terrorist attacks on cultural values as expressed in books

**DOI:** 10.1371/journal.pone.0311095

**Published:** 2024-11-22

**Authors:** Daniel Hart, I-Ming Chiu, Richard Epstein

**Affiliations:** 1 Prevention Science, Rutgers University, Camden, New Jersey, United Sates of America; 2 Department of Economics, Rutgers University, Camden, New Jersey, United Sates of America; 3 Department of English, Rutgers University, Camden, New Jersey, United Sates of America; University of KwaZulu-Natal College of Health Sciences, SOUTH AFRICA

## Abstract

We assessed the effects of the 9/11/2001 terrorist attacks on the salience of moral words and phrases in the United States, Great Britain, and Spanish-speaking countries, focusing particularly on those phrases related to authority and loyalty. Our predictions were that the 9/11 attacks would increase the salience of authority phrases suggestive of disorder (“chaos”, “disobedience”, etc, labeled *authority-vice*) and decrease words and phrases suggestive of organization (“hierarchy”, “obedient”, etc: *authority-virtue*) in books published in the United States. Similarly, we anticipated that the salience of phrases consistent with fidelity to members of one’s social group (“allegiance”, “one for all”, *loyalty-virtue*), would decrease, and those suggestive of social corrosion (“betrays”, “back stabs”, *loyalty-vice*) would increase. To test these predictions, we calculated the relative frequency of authority-vice, authority-virtue, loyalty-vice, and loyalty-virtue phrases, as well as those associated with other moral values, in books published in the U.S., Great Britain, and Spanish-speaking countries for each year between 1960 and 2019. A Bayesian structural time-series approach for each type of phrase provided additional support for the hypotheses for books published in the United States. Descriptive analyses suggested that the period following 9/11 was characterized by a deceleration in historical trends toward increasing use of moral vocabulary in published books. We discuss the implications of our findings for the measurement of cultural values and the impact of terrorism events on moral foundations and suggest that the encoding of these value shifts in texts is one way in which cultural effects are sustained.

## Introduction

Books serve many purposes, among them the transmission of knowledge and shaping of values. As Ong puts it in his classic work, “More than any other single invention, writing has transformed human consciousness” [1]. For example, Martin Luther so deeply believed in the power of reading the Bible to guide religious and ethical life that he advocated universal literacy so that each believer could relate directly to God’s word in text [2]. Authors of fiction frequently see themselves as elucidating moral issues [3]. Psychologists who study the effects of reading often focus on the consequences for ethical life [4]. The effects of reading and literacy for populations are better understood for economic development [2] than they are for moral development, but it seems likely that moral themes that pervade books both reflect and influence their readers. In this study, we focus on the moral language of books as it shifts in relation to a terrorist event and speculate about the effects of these deflections on the cultures in which they occur.

As is widely known, on September 11, 2001, terrorists hijacked four airliners. Two of them crashed into the World Trade Center towers in New York City, causing enormous damage and death to thousands of workers. A third plane crashed into the Pentagon, causing the partial collapse of the building and numerous deaths of civilians and military personnel. The hijackers of the fourth plane were thwarted by the passengers and it crashed in a rural field near Shanksville, Pennsylvania. Many of the immediate consequences of the attacks for the American public have been explored. For example, psychiatric research found that symptoms of post-traumatic stress disorder were more common among those exposed to the attack [5]. Generally, Americans were worried by the prospect of more attacks, with a majority reporting concern that they, their family members, or their friends could be injured in a terrorist attack [6].

Although some of the effects of 9/11 were short-lived, others were persistent [7]. Almost all (93%) Americans over the age of thirty surveyed in 2021 claimed to remember exactly where they were when the terrorist attacks occurred [7]. In 2016, 75% of Americans reported that the attacks were among the top historical events of their lifetimes. Finally, protecting the nation against terrorism has remained a top policy priority for Americans since 2001 [7].

A recent meta-analysis suggests two kinds of moral values were likely responsive to the 9/11 attacks. Godefroidt [8] integrated the findings of the effects on political attitudes of terrorist events reported in studies conducted between 1985 and 2020 and found that outgroup hostility, political conservatism, and “rally-‘round the flag” values tended to increase. This pattern is consonant with theory that the experience of threat and fear resulting from exposure to terrorism events can heighten concern with societal stability and order [9, 10]. For example, Hetherington and Suhay [11] used data from two national surveys to demonstrate that authoritarian attitudes were higher following the attack than before, and that this response was most pronounced among those who were low in authoritarianism prior to the attack. Echebarria-EChabe and Fernandez-Guede [12] used survey data concerning authoritarianism collected in Spain just prior to and following the Madrid railway terrorist attack in 2004 to infer that the effect was to increase authoritarianism, conservatism, and to lower attachment to liberal values. This work suggests that terrorist attacks may make salient moral concerns with societal chaos, the breakdown of social order, and the dissipation of respect for authority.

Second, terrorism, by increasing fear, can deepen intra-group favoritism and inter-group friction. As already noted, this effect is apparent in Godefroidt’s [8] meta-analysis of hundreds of studies of the effects of terrorism on political values. For example, Kam and Kinder [13] argued that ethnocentrism became more salient following the attack on the World Trade Center. Kam and Kinder used a longitudinal sample from the National Election Studies; in 2000, ethnocentrism was assessed by asking participants to compare their own racial/ethnic group on dimensions of intelligence and character to other racial/ethnic groups. In 2000 and in 2002, the same participants were asked a variety of questions concerning national security and military spending. The analyses indicated that ethnocentrism was more predictive of attitudes in 2002 than in 2000. The authors interpreted this pattern as an indication that ethnocentrism became more important in shaping the attitudes of Americans following the attack on the World Trade CenterEchebarria-Echabe and Fernandez-Guede [12] leveraged surveys collected immediately before and after the Madrid railway terrorist attack to identified increases in prejudice against members of outgroups. This pattern suggests that the moral salience of fidelity with one’s fellow group members may have increased following the attacks.

Significant terrorism like the 9/11 attacks have geographically disbursed effects. For example, Schüller [14] reported that one effect of the 9/11 attacks in the United States was to increase anti-immigration attitudes among Germans. Similarly, Bömelt, Bove, and Nussio [15] found that terrorism events in one country raised anti-immigrant sentiment in neighboring European countries. There was reason to imagine, then, that the cultural effects of the 9/11 attacks would be evident in countries besides the United States, though these effects may be diminished by geographical and cultural distance [16].

We sought to determine whether the terrorist attacks effected change in cultural values, particularly those related to authoritarian and inter-group moral concerns. Following Michel and colleagues [17], we used a culturomics approach, assessing facets of culture and their change over time by charting the salience of words in millions of published texts digitized by Google. Inferring cultural shifts from words in digitized books has two risks. The first of these is that digitization is imperfect as a consequence of typographical similarities that may result in errors. For example, in some type faces, *s* and *f* are very similar, and as a consequence the word *sail* might be digitized as *fail*. [18] Nonetheless, the error rate in digitization of words for the period relevant for this study is estimated to be ~2% [17].

The second, and perhaps most significant challenge to inferring cultural trends, is that the composition of the sample of books digitized by Google shifts over time; the fraction of words drawn from science books varies from decade to decade, for example [18]. In this study, we compare trends across corpora as a means of testing the robustness of our results. We supplement the analyses of the effects of the 9/11 attacks evident in three corpora with tests using a corpus provided by Google that draws only from works of fiction, because word frequencies in this corpus are unaffected by year-to-year variations in the fraction of nonfiction books contributing words to the corpus. [19] and of the effects of two other unexpected attacks.

One example of the culturomics approach is offered by Acerbi and Sacco [20] who studied the salience of words related to self-control (control, modulate, manage, resist) and self-indulgence (allow, coddle, comfort, pamper) over the course of the 20^th^ century. They found that words reflective of self-indulgence diminished from 1900 to about 1960, and then began to increase. The authors argued that this change reflected the rapid expansion of consumerism and the valuing of self-expression in American culture that had been identified as occurring at the same by historians and cultural analysts [20]. The trends identified by Acerbi and Sacco suggest that the analyses of the salience of words published in books can capture important changes in culture.

Mitts [21] demonstrated that right-wing vocabulary increased in books published in Israel because of the second Intifada. To identify right-wing vocabulary as used in Israel, Mitts drew words and phrases from liberal and conservative Israeli party platforms and augmented this initial dictionary with phrases drawn from the Hebrew version of Wikipedia. Mitts used the peak of casualties during the second Intifada to bifurcate the cultural history of Israel in the early 21st century and found that right-wing vocabulary increased in the era after the second Intifada.

Our approach parallels that of Michel et al. [17], Acerbi and Sacco [20] and Mitts [21]. We assessed the relative frequency of a set of words corresponding to moral values. Instead of assessing the impact of politically influenced censorship or a sustained period of violence on frequency over time, we investigated the consequences of a terrorism event on the historical trajectory of classes of words.

Haidt and his colleagues have proposed that five clusters of moral values can be identified in cultures around the world [22]. These five clusters, or foundations, are morality as fairness, signaled by the concern for justice and reciprocity; morality as loyalty, centered around values of loyalty, in-group commitments, and patriotism; morality of harm and care, revealed in empathy and nurturance; morality of authority, reflected in obedience to and respect for hierarchies; and, finally, morality of sanctity, reflected most clearly in concerns for contamination and carnality. Haidt and his colleagues have demonstrated that the moral foundations can be distinguished through different kinds of measurement, appear in different cultures, and add to predictions of a range of theoretically related attitudes and behaviors [22].

Graham, Haidt, and Nosek [22] suggested that the importance of a moral foundation is revealed by its salience in language. Individuals with conservative worldviews, Graham and colleagues predicted, would likely be oriented towards obedience and respect for authority. In one study, they predicted that ministers of conservative denominations would be more likely than ministers of liberal churches to give sermons that reflect an orientation towards authority and tested this hypothesis by coding sermons for the presence of words chosen as representative of each of the moral foundations. For example, words like “respectful”, “obedient”, “hierarchical”, and “traditional” were selected as prototypical of a moral value orientation towards the moral foundation of authority. Table 1 provides examples of words and phrases connotative of the moral foundations of authority and loyalty. Graham, Haidt, and Nosek found that words conveying an orientation towards the moral foundation of authority were more common in sermons prepared by ministers in conservative traditions than in sermons given by liberal ministers.

**Table 1:** Phrases used in moral foundation dictionaries to code authority and loyalty.

	Authority	Loyalty
Virtue	servant	kin
	social order	organization
	supervisor	sacrificial
	servants	war
	honoring	allegiances
	bow down	group
	presidential	cliques
	obediently	ally
	revered	groups
	acquiesce	herds
	take up arms	sacrifice
	top gun	uniter
	governs	brothers in arms
	commandments	allegiance
	hierarchy	factions
	master	unite
	mentors	country
Vice	disobedience	heretics
	overthrows	treachery
	rebelling	outgroup
	rebel	rebellions
	unlawfully	outsiders
	rebellion	backstabbers
	refuses	against us
	heretical	heresies
	dissenting	betray
	disarray	backstabbed
	mutinied	cheats on
	dishonouring	backstabbing
	subverted	traitor
	transgresses	unfaithfulness
	overthrown	infidel
	renegades	deserting
	dissenters	backstabber

We proceeded in five steps.

First, the sensitivity of the culturomics approach to the 9/11 attacks was assessed by investigating the relative frequency of the word “terrorism” before and after 2001. Following Michel et al. [17], the prediction was that the frequency of “terrorism” in published books ought to increase, just as Michel et al found that “Tiananmen” was more commonly found in books (except those published in China) following the Tiananmen Square demonstration.

In the second step, changes in the relative frequency of words expressing fear before and after the attacks were examined. As described earlier, hypotheses linking terrorism to increases in authoritarianism and inter-group conflict typically posit that heightened fear is an intervening mechanism. Evidence that fear words in published books increased following the attacks would be consonant with the hypothesis that 9/11 terrorist attacks increased the salience of fear in English and Spanish-speaking countries, which precipitated shifts in the prominence of the moral foundations related to authority and loyalty.

Third, we examined the salience of moral language in general before and after the 9/1ll attacks.

Fourth, we explored support for our hypotheses that the 9/11 terrorist attacks would affect the salience of words and phrases related to authority and loyalty.

Finally, we provide descriptive analyses of the relative frequency of words corresponding to the three other moral foundations before and after the 9/11 attacks.

## Methods

Using optical character recognition (OCR), Google has digitized more than 9 million books published in the U.S., more than 2 million published in Great Britain, and more than 1 million published in Spanish. The books included for digitization were selected for inclusion in a database based on the high quality of the OCR data and the presence of metadata such as location and year of publication [17]. In supplementaryco analyses, we used a fourth corpus of words provided by Google, drawn from books published in English from works of fiction. We used data from books in this database published between 1900 and 2019. These data are publicly available and can be downloaded from http://books.google.com/ngrams/datasets.

In these data sets, a string of characters that is uninterrupted by a space or punctuation is called a 1-gram. With some exceptions (e.g., numbers, punctuation, hyphenated words), a 1-gram corresponds to an English word. A string of characters interrupted with a space is a 2-gram, and so on. The Google data sets contains 1-, 2-, 3-, 4-, and 5-gram character strings.

To identify phrases corresponding to the five moral foundations we used dictionaries developed by Graham, Haidt, and Nosek [22] and revised by Frimer et al. [23]. This dictionary has been translated into Spanish by Bruegger and Borer [24]. These dictionaries contain single words and short phrases for the virtue (positive) and vice (negative) poles of each moral foundation (see Table 1; the dictionary has between 115 and 388 phrases for each combination of foundation and pole). We matched the phrases in these dictionaries to the 1-gram, 2-gram, 3-gram, 4-gram, and 5-gram phrases in the Google database. For each year between 1900 and 2019, we used the ngramr package [25] to query the Google Books Ngram Viewer to obtain the relative frequency of n-grams matching authority-vice phrases in the moral foundation dictionary (relative frequency is the number of times a phrase appeared in a year divided by the total number of phrases of the same gram length available for that year). The identical process was used for vice and virtue phrases for the other four moral foundations. We also calculated the relative frequency of the word “terrorism” for books published in English in the U.S. and Great Britain and “terrorismo” for books published in Spanish. Finally, we abstracted the frequency of four fear words in English (“afraid”, “frightened”, “scared”, “terrified”) and three in Spanish (“atemorizado”, “asustado”, “aterrorizado”).

The procedure suggested by Younes and Reips [18] was used to calculate summary measures for the moral foundations and the fear words. Younes and Reips point out that raw frequencies of words and phrases in the Google corpora have substantially different means and standard deviations. For example, in books published in the United States between 1900 and 2019, the word “afraid” has a mean frequency of 0.000023 and a standard deviation of 0.0000078 compared to a mean of 0.000007 and a standard deviation of 0.0000017 for the word “frightened”. Averaging the raw frequencies for these two words results in an index in which the frequency for “afraid” dominates. A better procedure is to standardize the frequency of each word and phrase prior to averaging, which results in a summary measure in which each word or phrase contributes equal weight. We have computed averages of z-scores for each of the moral foundation scores (authority-virtue, authority-vice, loyalty-virtue, loyalty-vice) and the fear words.

Younes and Reips [18] also note that the Google word corpora may be expanding with more words being used in published books which would have the result of each word or phrase declining in frequency over time. To adjust for this possibility, Younes and Reips recommend averaging the z-scores for frequencies of very common words in a language (for example in English “the”, “a”, etc) and subtracting this mean from the indices of interest. We have used this procedure to calculate summary scores for the moral foundations and the fear words to minimize the impact of historical changes in the word corpora on the trends of interest. Younes and Reips report that this index appears to provide the most sensitive tests of conceptual change measured through word frequencies in the Google word corpora.

## Results

**Fig 1:**
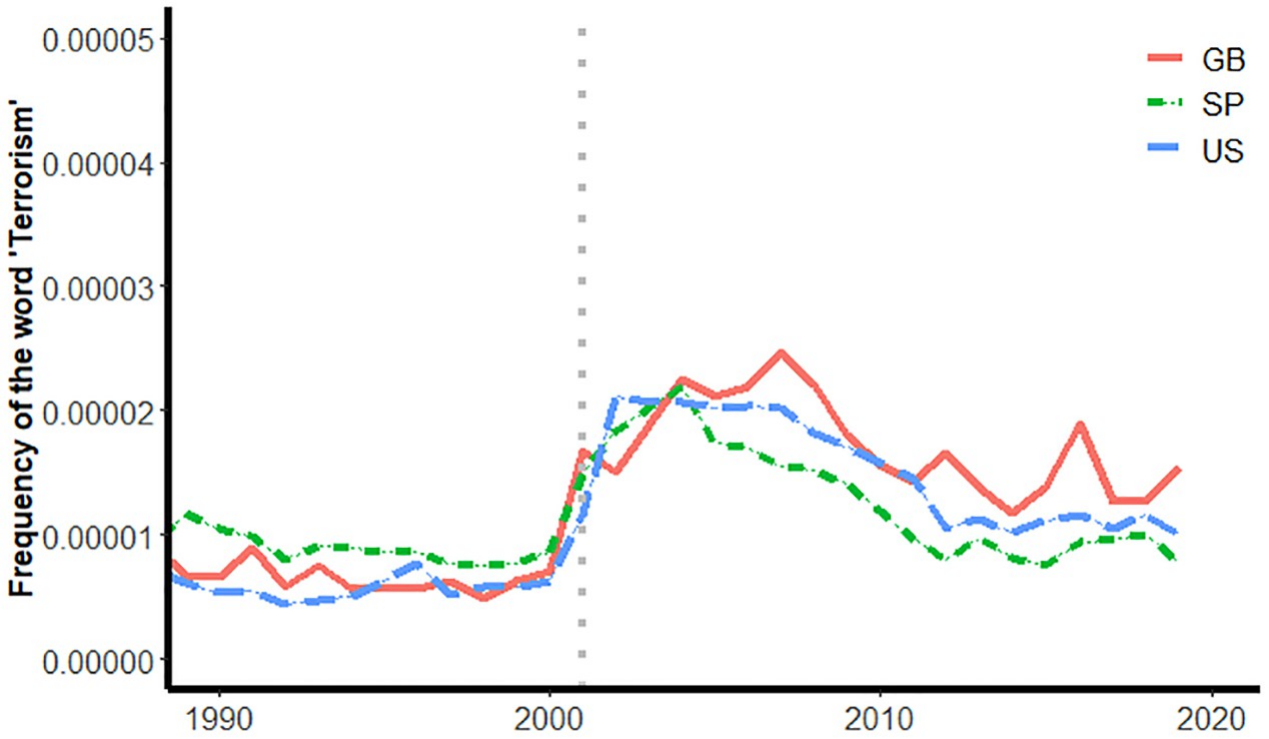
Frequency of the word ‘Terrorism’ in books published in the United States, Great Britain, and in Spanish-speaking countries, by year.

Fig 1 depicts the salience of the word “terrorism” in books published in the United States, Great Britain, and Spanish-speaking countries before and after the 9/11 attacks.

The trends evident in Fig 1 indicate that the word terrorism becomes two to three times more common in books published in English and in Spanish in the years immediately after the 9/11 attacks than was true just prior (a vertical gray dotted line in Fig 1 is positioned to intersect the x-axis at 2001, the year of the attacks). The increased frequency of “terrorism” in books has been maintained for more than a decade. This trend supports the claim that word salience in published books–in this instance, the frequency of the word “terrorism”–is sensitive to significant world events.

**Fig 2:**
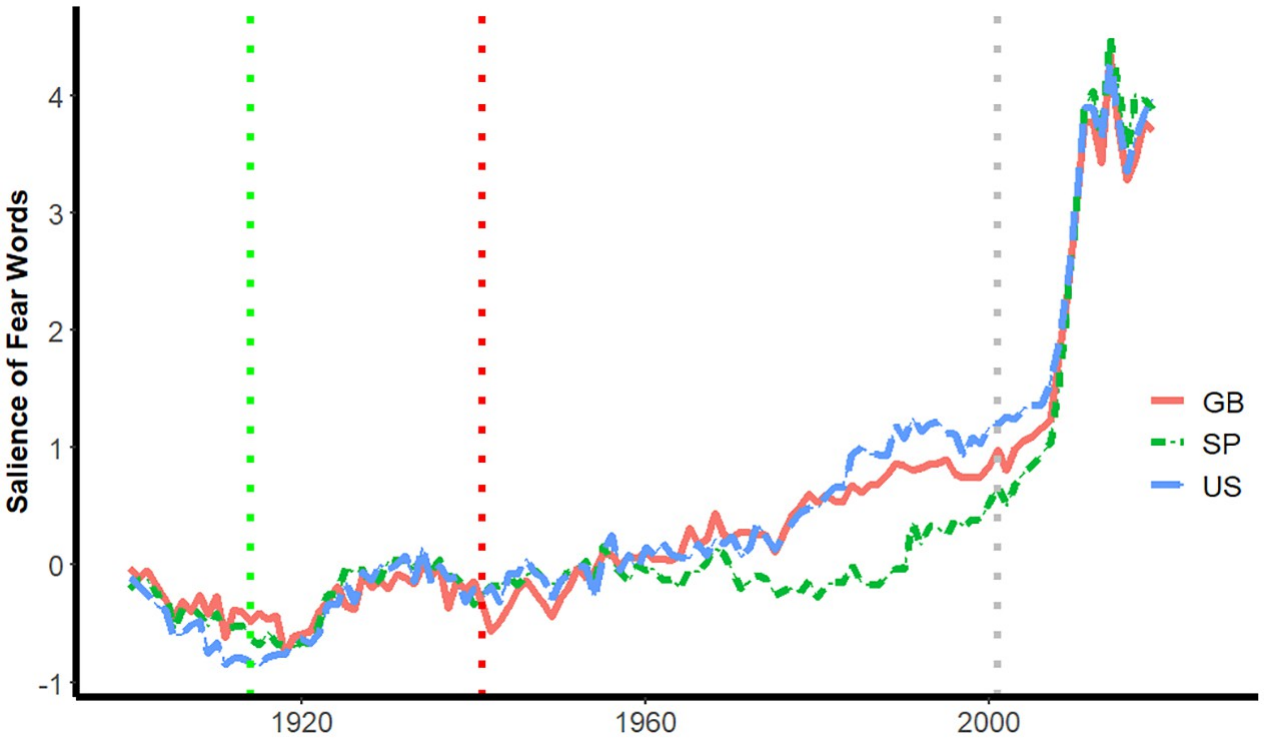
Salience of fear words by year by country.

Fig 2 depicts the salience of fear words in books published in the U.S., Great Britain, and in Spanish-speaking countries by year. The trends in Fig 2 suggest that immediately following the attacks in 2001, corresponding to the gray dotted vertical line, there was a slight change in the salience of fear words, followed by a rapid acceleration in salience beginning in 2006. The trends in Fig 2 are consistent with an interpretation that the terrorist attacks of 9/11 increased the salience of fear among authors writing books in English and in Spanish.

Fig 2 also has dotted lines corresponding to the onset of World War I (1914) and the Pearl Harbor attack (1941). We added these to provide some context for the lag between an event and the increase in fear words and the magnitude of change evident in fear words following the 9/11 terrorist attacks.

As to the lag, it appears that increases in fear words were evident in English and Spanish 3–5 years following the onset of the world wars, approximately the interval at which fear words increase dramatically following the 9/11 attacks. Notably, however, the magnitude of increase is dramatically larger following the 9/11 attacks than that observed following either world war. It’s likely that the much larger impact on fear words resulting from the 9/11 attacks in comparison to that of Pearl Harbor and the onset of World War II reflects differences in media, elite representations, perceptions of the nature of the threat, and national identifications [26].

**Fig 3:**
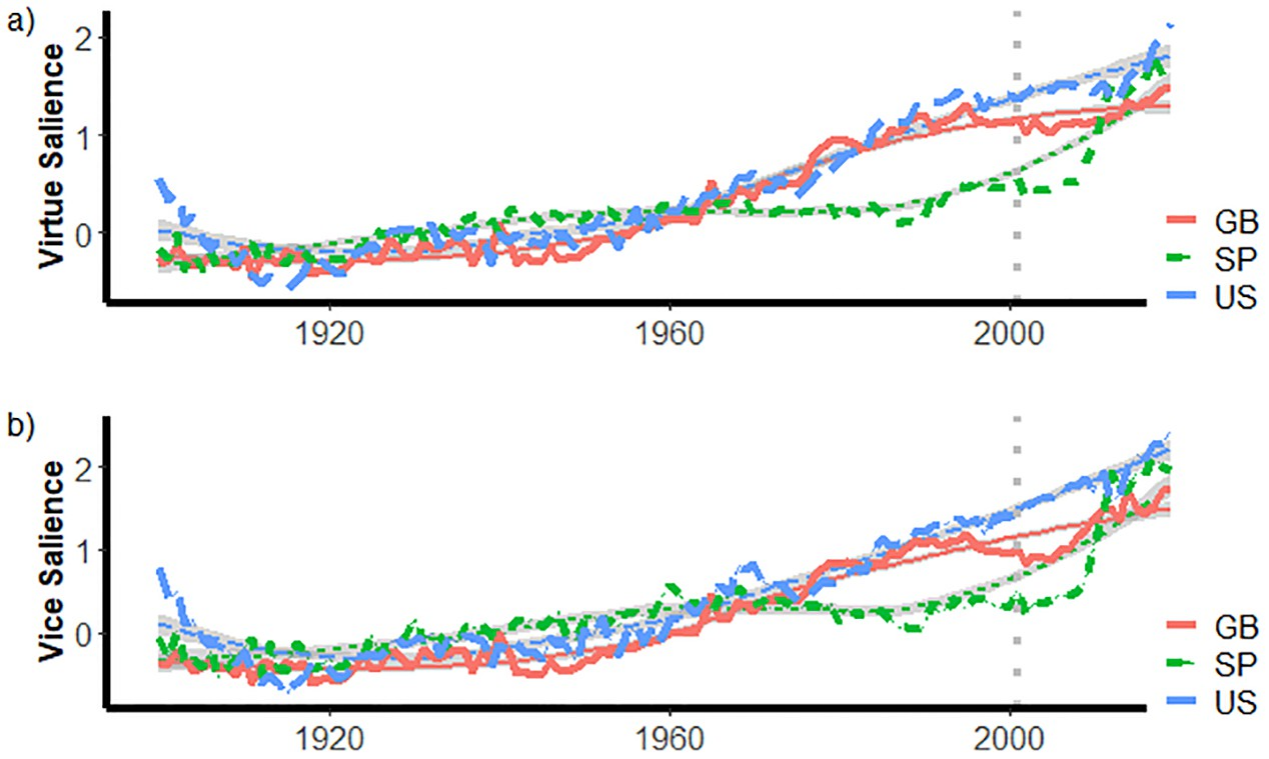
Salience of virtue and vice phrases by year by country.

Fig 3a presents the average of salience of virtue phrases by year and by country and Fig 3b presents the average of salience of vice phrases. Together, 3a and 3b indicate that moral language as characterized by the dictionaries for the moral foundations generally increased over the course of the 20th century.

In all three corpora, increases in the salience of the virtue words slowed in the 1990s and then began increasing again in the second decade of the 21st century.

The smooth lines in Fig 3a are loess curves for each country. Loess curves are derived from locally-weighted regressions in which each point on the curve is estimated based on its closest neighbors. The benefit here is that these lines illustrate trajectories of virtue salience with year-to-year perturbations minimized. The loess curves are not formal statistical estimates of counterfactual trajectories in the absence of the 9/11 attacks—these are provided later—but they are an aid in the graphical display of deflections from smoothed trends.

In Fig 3a, it is evident that there are substantial departures between the observed trajectories and the loess curves for all three corpora following the 9/11 attacks. The gap is largest for books published in Spanish and is perhaps evident just prior to 2001. A similar trend is evident for books published in Great Britain. The observed salience and that depicted in the loess curves for the United States are similar for the first years following the 9/11 attacks, but then diverge sharply, as the actual salience is much lower than what is depicted in the loess curve.

Fig 3b suggests that the salience of vice phrases increased steadily from 1950 through 2019 in books published in the United States. In the other two corpora, the salience of vice phrases was relatively constant from approximately 1980 through 2000, and then began to increase.

Once again, loess curves sharpen an understanding of post-9/11 trends for each country. The divergence is substantial between observed and loess curves for books published in Great Britain; the gap begins before 2001 but grows larger in the years immediately following the attacks. In books published in the United States and in Spanish, the observed salience is first below, then later above the salience depicted by the loess curves.

To check whether these trends are artifacts of the change in the composition of the books scanned by Google, we also graphed the trends for each moral foundation virtue and vice for fiction published in English. As noted earlier, some researchers [19] have noted that the English fiction corpus is unaffected by variations in the fraction and nature of non-fiction books that characterize the other corpora. We present these graphs in the supplemental materials ([https://osf.io/brzjw/?view_only=083ffbdddd8d44d6867c79cae9006ea8], S1 and S2 Figs); generally, these graphs are consistent with Fig 3a and 3b. The corpus drawn from fiction published in English also suggests that the salience of virtue phrases stopped increasing around the end of the 20th century.

**Fig 4:**
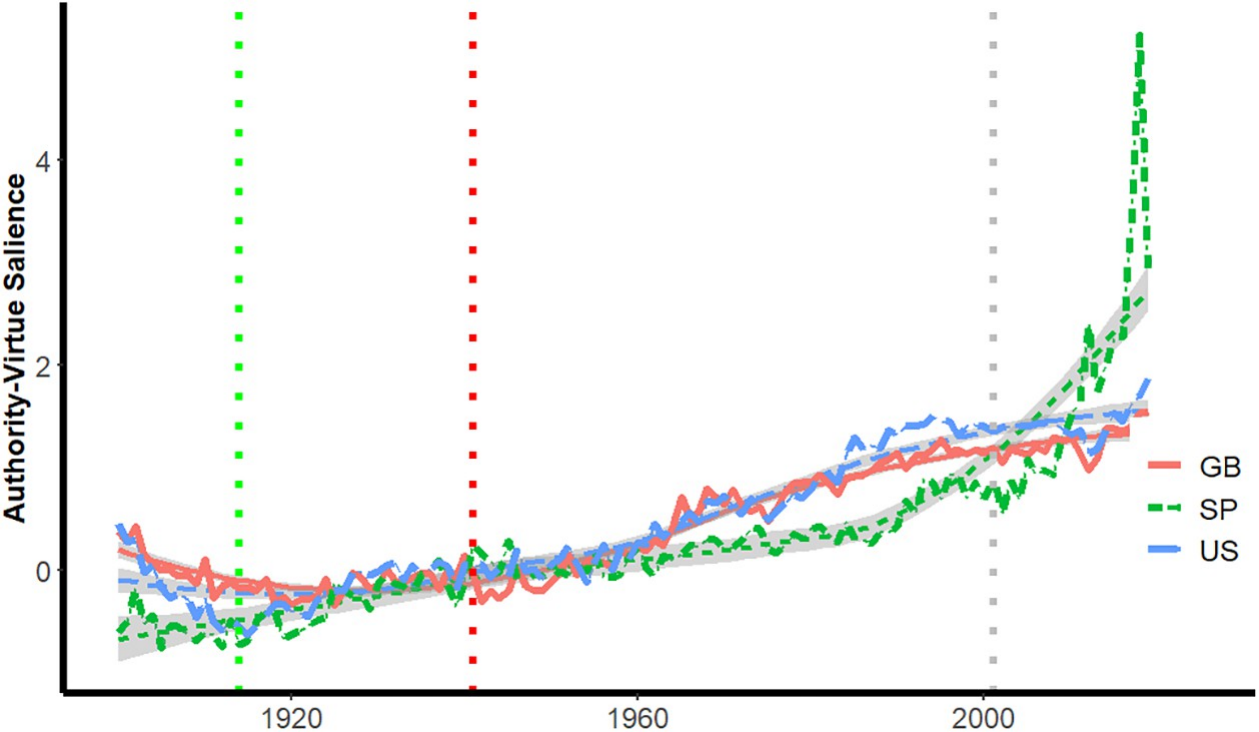
Salience of authority-virtue phrases by year.

**Fig 5:**
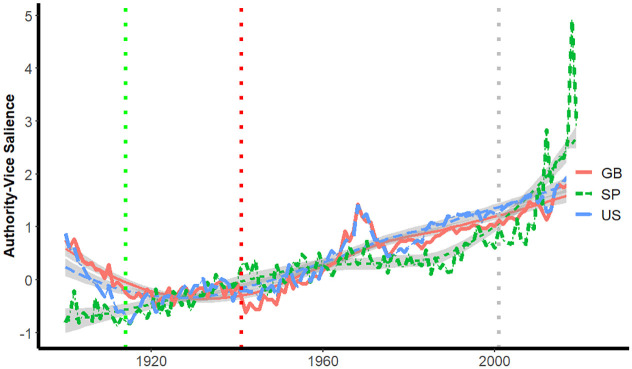
Salience of authority-vice phrases by year.

Figs 4 and 5 depict the salience of authority-virtue and authority-vice phrases for years between 1900 and 2019 for the three corpora. The dotted vertical line intersects the x-axis at 2001, corresponding to the year of the attack on the World Trade Center.

Once again dotted lines are inserted for the onset of World War I and the attack on Pearl Harbor, and loess lines provide smoothed trajectories for each country. In all three corpora, there are substantial depressions beginning close to 2001 in observed salience relative to that suggested by the loess lines; these depressions appear first in the Spanish corpora, then, in books published in Great Britain and finally in U.S. books.

Fig 5 depicts the salience of authority-vice phrases in the three corpora.

Fig 5 provides observed and loess-smoothed trajectories for Authority-Vice words for the three corpora. There is less evidence of substantial divergences near 2001 between observed and smoothed trajectories than was evident for authority-virtue phrases. The spikes in authority-vice (Fig 5) and authority-virtue (Fig 4) salience observed in all three corpora for the most recent decade are consistent with the dramatic increases in political populism discussed in the introduction.

**Fig 6:**
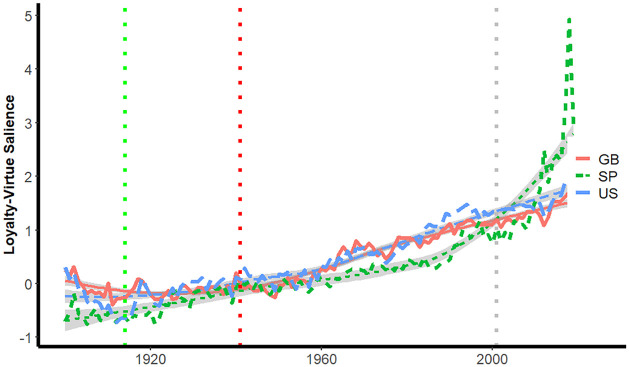
Salience of loyalty-virtue phrases by year.

The trends for loyalty-virtue phrases in Fig 6 are consistent with the hypothesis that the 9/11 attacks triggered decreases in the corpora for books published in the U.S. and Great Britain, signaled by the depression of the observed salience lines below the smoothed loess lines. There is little evidence for an effect for the Spanish corpora.

**Fig 7:**
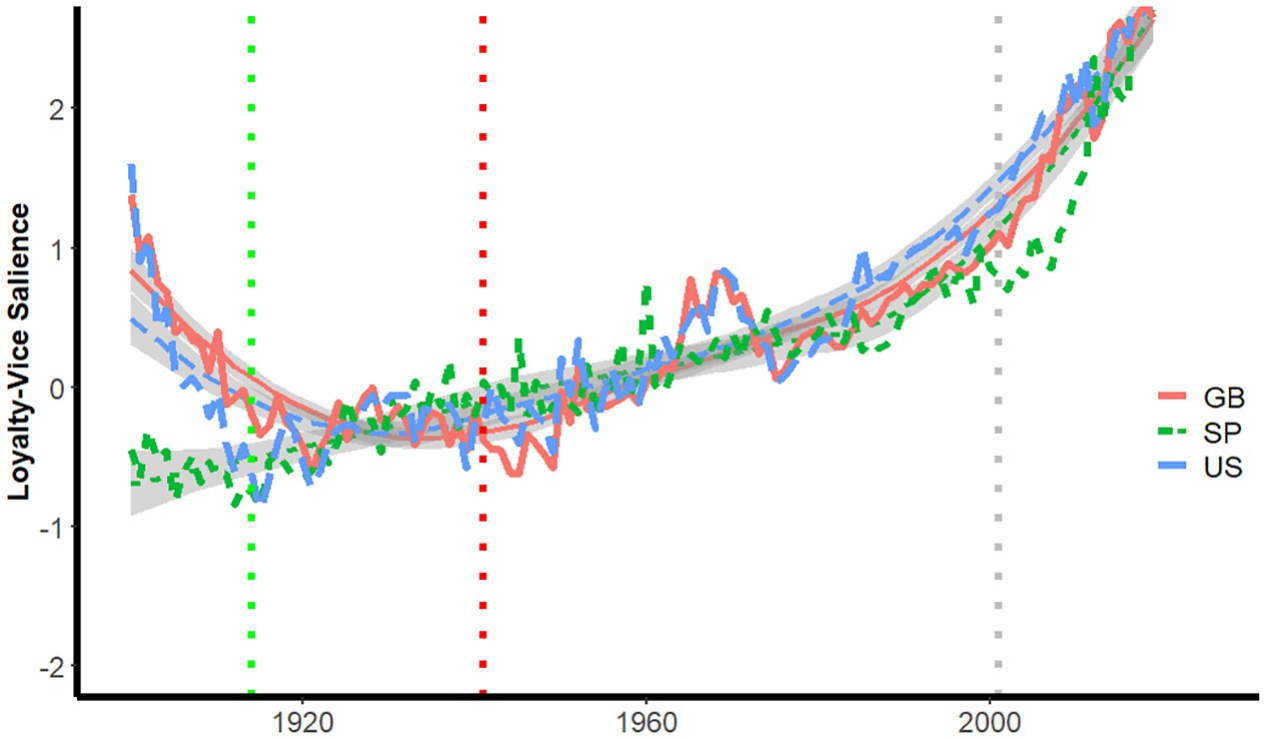
Salience of loyalty-vice phrases by year.

Fig 7 suggests differences in trajectories in the increases in salience of loyalty-vice phrases in the three corpora. For books published in the U.S. and Great Britain, following the 9/11 attacks observed salience exceeds that depicted by the loess curve; n. The trajectory of loyalty-vice phrases in Spanish-language books follows a different path, with a substantial increase in salience following World War II with general stability thereafter–even following the 9/11 terrorist attacks, followed by a sharp increase.

It is the difficulty in this study of confident visual detection in graphs of changes in time series as a function of a hypothesized impact–the 9/11 attacks in this research–that makes useful a *synthetic control analysis*. Synthetic control analysis was originally developed to assess the impact of interventions on large units such as countries and offers advantages to traditional time-series analysis [27]. In the analyses that follow, we use a synthetic control approach to model the expected trajectory of authority-virtue, authority-vice, loyalty-virtue, and loyalty-vice salience scores if the 2001 terrorism attacks had not occurred. These modeled trajectories are then compared to the observed trajectories to infer the impact of the terrorism events.

Specifically, we used the Bayesian structural time-series approach described by Brodersen, Gallusser, Koehler, Remy, and Scott [28]. This causal impact is formulated as the difference between the time series that is observed and the time series that would have obtained in the absence of the intervention. To estimate the time series that would have occurred in the absence of the intervention, information is drawn from the time series prior to the intervention, and, when possible, from correlated time series that are unaffected by the hypothesized impact (for an example in another domain, see Bruhn et al. [29] for an application of this method to time series of health data to infer vaccination effectiveness).

In the analyses that follow, we used yearly per capita GDP as a control variable. Table 2 presents the correlations of the time series of interest in this paper with the time series for per capita GDP for the United States and Great Britain. Correlations are high, a fact to be expected as Figs 3–6 all suggest that the saliences of the moral foundation phrases of interest are correlated with year, as is per capita GDP. Particularly useful for the interrupted time-series analysis are variables associated with the predictor that are largely unaffected by the hypothesized impact. In general, the effect of terrorism attacks on per capita GDP is transient or insignificant [30]. The consequences of the 9/11 attacks on the economy of United States had a substantial, short-term impact on economic forecasts, but that effect dissipated very quickly [31]. The correlations of per capita GDP with the salience indices, combined with per capita GDP’s substantial, but not total independence from the 9/11 attacks make it an excellent covariate [28] for developing the synthetic control.

**Table 2:** Correlations among time series for authority and loyalty salience for three corpora and GDP.

	Variable	*(1)*	*(2)*	*(3)*	*(4)*	*(5)*	*(6)*	*(7)*	*(8)*	*(9)*	*(10)*	*(11)*	*(12)*	*(13)*	*Mean*	*SD*
US	*Authority-Virtue (1)*														0.48	0.69
	*Authority-Vice (2)*	0.96													0.48	0.75
	*Loyalty-Virtue (3)*	0.99	0.96												0.49	0.67
	*Loyalty-Vice (4)*	0.89	0.95	0.91											0.48	0.88
	*GDP (5)*	0.86	0.88	0.92	0.96										26096.5	19126.1
GB	*Authority-Virtue (6)*	0.97	0.92	0.95	0.82	0.79									0.33	0.6
	*Authority-Vice (7)*	0.96	0.93	0.93	0.84	0.81	0.99								0.19	0.65
	*Loyalty-Virtue (8)*	0.96	0.91	0.95	0.83	0.83	0.99	0.98							0.44	0.61
	*Loyalty-Vice (9)*	0.94	0.93	0.93	0.91	0.91	0.96	0.97	0.96						0.07	0.63
	*GDP (10)*	0.83	0.85	0.88	0.93	0.98	0.74	0.75	0.79	0.86					20241.5	16635.8
Spanish	*Authority-Virtue (11)*	0.75	0.78	0.79	0.82	0.77	0.72	0.72	0.73	0.79	0.68				0.24	0.37
	*Authority-Vice (12)*	0.72	0.77	0.75	0.75	0.52	0.71	0.71	0.71	0.75	0.4	0.94			0.24	0.45
	*Loyalty-Virtue (13)*	0.85	0.86	0.88	0.86	0.88	0.85	0.84	0.86	0.88	0.82	0.96	0.91		0.24	0.54
	*Loyalty-Vice (14)*	0.64	0.7	0.69	0.74	0.56	0.62	0.62	0.63	0.7	0.48	0.94	0.94	0.9	0.24	0.53

We used per capita GDP for the U.S. and for Great Britain to construct the synthetic controls for all analyses. In each analysis, the time series of interest was combined with the time series for per capita GDP in a state-space time-series model. We used the program CausalImpact developed by Brodersen, Gallusser, Kohler, Remy, and Scott [28] to implement the Bayesian structural time-series analysis. The program takes as inputs for the estimation of a synthetic control the two per capita GDP time series for the period 1960–2019 and the time series for the salience score from post-World War II period 1960–2001, and then compares the resulting synthetic control time series with the time series for the salience score observed for the period between 2002 and 2019. We use the period from 1960 to 2001 and compare that to the time series from 2002 to 2019. The period from 1960 to 2001 covers a time period without global wars; the period from 2002 to 2019 covers the span following the 9/11 attacks for which ngrams have been made available by Google. The analysis allows an estimation of the difference in the time series before and after the event, which is defined as the average of the differences between the observed data points and those in the synthetic control Confidence intervals are computed in the analysis for each estimate.

**Fig 8:**
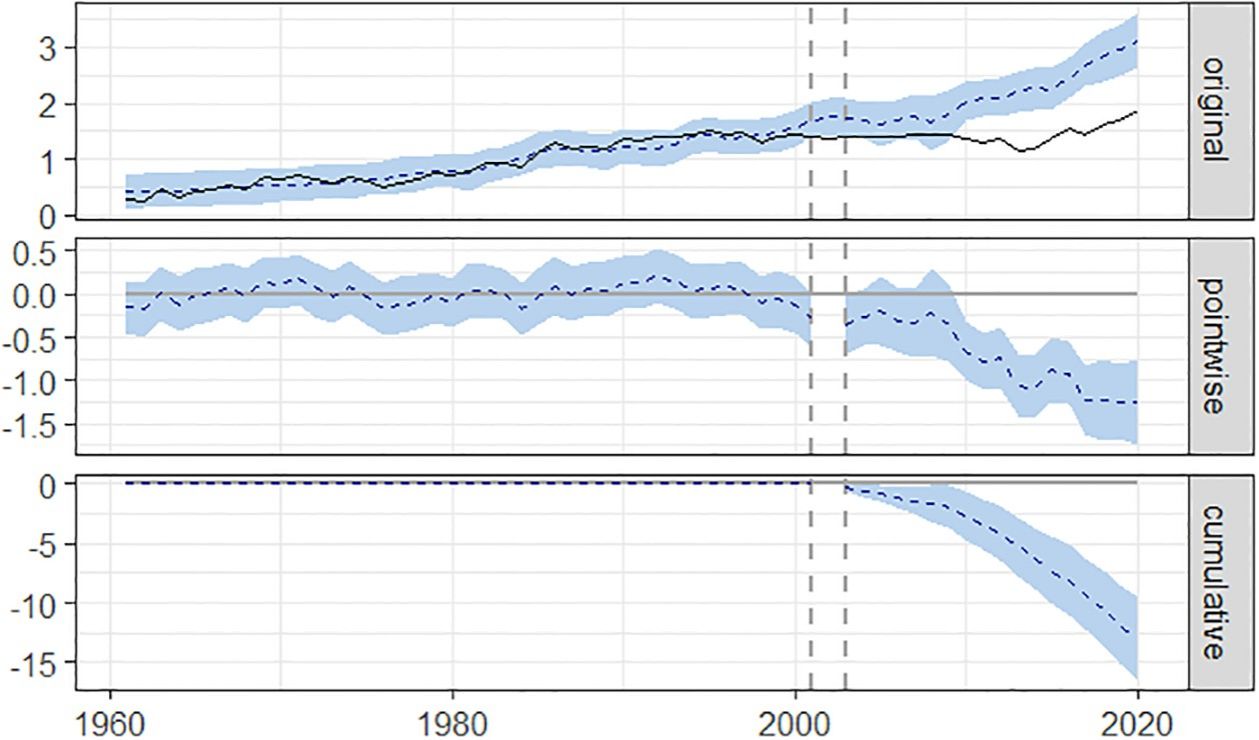
Estimated impact of the 9/11 terrorism attacks on authority-virtue word salience in the U.S.

Fig 8 illustrates the information provided by the analysis. In the top panel of Fig 8, the solid line illustrates the observed trajectory of authority-virtue word salience in the U.S.; the dotted line in the top panel traces the counterfactual path estimated by the procedure. In the middle panel, the dotted line traces the divergence between the observed and modeled trajectories for each year; as is evident in this panel, the divergence between modeled and observed expands in the 21st century, consistent with hypotheses. Finally, the bottom panel illustrates the cumulative effect of the differences between modeled and observed for the post-impact (post terrorism attacks) observation period, 2002–2019.

The graphs indicate that the salience of authority-virtue phrases was depressed substantially below what the synthetic control model predicts; the latter is based on the pre-2001 trajectory combined with information from GDP data. The depression of authority virtue phrases is consistent with our hypothesis.

Table 3 presents the estimates from the CausalImpact analysis for each moral foundation virtue and vice for each country. The column labeled *Actual* in Table 3 presents the average observed trend. The *Estimate* column provides the synthetic control estimate in the absence of the intervention (the 9/11 terrorist attacks). The *Causal Impact* column is the difference between the actual effect and the synthetic control estimate. The confidence intervals for the synthetic estimate are provided in the next two columns. If the average observed (*Actual)* is outside of the confidence intervals for the synthetic control, then it is considered statistically significant.

**Table 3:** Observed values, synthetic control estimates, causal effects, with confidence intervals, by country and moral foundation.

				Estimated Effect			
	Foundation	Actual	Estimate	Causal Effect	Lower Bound	Upper Bound	p
US	Authority-Vice	1.62	1.63	−0.01	1.40	1.86	0.46
	Authority-Virtue	1.43	2.15	−0.72	1.96	2.36	0.00
	Care-Vice	2.09	2.42	−0.33	2.16	2.67	0.01
	Care-Virtue	1.89	2.56	−0.67	2.29	2.83	0.00
	Fair-Vice	1.70	2.01	−0.31	1.82	2.20	0.00
	Fair-Virtue	1.16	2.28	−1.12	2.08	2.49	0.00
	Loyalty-Vice	2.17	1.62	0.55	1.37	1.85	0.00
	Loyalty-Virtue	1.53	2.04	−0.50	1.84	2.23	0.00
	Sanctity-Vice	1.86	2.25	−0.38	2.03	2.47	0.00
	Sanctity-Virtue	1.81	1.82	−0.02	1.60	2.02	0.44
GB	Authority-Vice	1.44	1.21	0.23	1.01	1.40	0.01
	Authority-Virtue	1.25	1.63	−0.38	1.46	1.79	0.00
	Care-Vice	1.95	1.95	0.00	1.73	2.16	0.49
	Care-Virtue	1.74	2.08	−0.34	1.86	2.30	0.01
	Fair-Vice	1.57	1.57	0.01	1.39	1.73	0.47
	Fair-Virtue	1.01	1.87	−0.86	1.68	2.06	0.00
	Loyalty-Vice	2.00	1.21	0.79	1.01	1.40	0.00
	Loyalty-Virtue	1.35	1.55	−0.20	1.40	1.70	0.01
	Sanctity-Vice	1.68	1.72	−0.04	1.54	1.89	0.34
	Sanctity-Virtue	1.62	1.41	0.21	1.24	1.56	0.01
SP	Authority-Vice	1.77	0.89	0.88	0.69	1.07	0.00
	Authority-Virtue	1.81	1.16	0.65	1.00	1.32	0.00
	Care-Vice	1.89	1.02	0.88	0.87	1.17	0.00
	Care-Virtue	1.77	1.19	0.58	1.02	1.35	0.00
	Fair-Vice	1.76	0.88	0.87	0.73	1.03	0.00
	Fair-Virtue	1.65	1.00	0.65	0.82	1.17	0.00
	Loyalty-Vice	1.81	1.11	0.70	0.96	1.26	0.00
	Loyalty-Virtue	1.88	1.36	0.52	1.18	1.54	0.00
	Sanctity-Vice	1.72	1.02	0.70	0.85	1.18	0.00
	Sanctity-Virtue	1.65	0.84	0.81	0.70	0.97	0.00

To refresh, our predictions were that authority-virtue and loyalty-virtue phrases would be depressed in books published in the United States, and that authority-vice and loyalty-vice phrases would increase. Two of the three remaining hypotheses, in addition to the one concerning authority-virtue just discussed, are supported by the estimates in Table 3. Relative to the synthetic control trajectories, and as predicted, loyalty-vice phrases increased (by an average of 34%) and loyalty-virtue phrases decreased following the 9/11 terrorism attacks (an average decrease of -26%). However, the observed values for authority-vice did not differ on average during the post-9/11 period from the values predicted for the synthetic control for books published in the United States.

Table 3 suggests that the salience for authority-virtue loyalty-virtue declined, and loyalty-vice increased, in books published in Great Britain, the pattern observed for books published in the United States. Finally, in books published in Spanish, Table 3 suggests that authority-virtue, authority-vice, loyalty-virtue, and loyalty-vice all increased relative to what was predicted by the synthetic control.

In the Supplementary Materials, we provide additional evidence of the robustness of the findings related to the hypotheses. As noted earlier, some researchers have suggested that analyses of Google’s fiction corpus may be less sensitive to the effects of the changing composition of samples of books included in the three corpuses used in this study that include works of fiction and non-fiction. In S1 Table we report the results of analyses that parallel those in Table 3 for authority and loyalty. As was true for the corpus of books published in the U.S., authority-virtue decreased significantly, and loyalty-vice increased, following the 9/11 terrorist attack. We also tested the impact of the attack on Pearl Harbor on the salience of authority and loyalty values as measured in words published in books in the U.S., comparing the trajectories of values between the end of the great depression (1929) and the attack (1941) and the period between 1942 and 1960. This set of analyses is reported in S2 Table and found that authority-vice and loyalty-vice words increased following the attack. In a final analysis, we compared the trajectory of authority and loyalty values in books published in Spanish in the decade before and after the Hipercor terrorist bombing in Madrid (S3 Table) and found that the trajectories of all four moral foundations were elevated following the attack. This latter pattern is identical with that obtained from the analyses before and after 9/11. Although the patterns are not identical across all the corpuses and time periods, there is considerable consensus suggesting that terrorist attacks are associated with predictable changes in cultural values as reflected in books published at different times and in different languages.

In the Introduction we noted that while the literature led to predictions concerning authority and loyalty moral foundations, these are only two of the five moral foundations proposed by Haidt and his colleagues [18]; the other three are care (care-virtue, care-vice), fairness (virtue and vice), and sanctity (virtue and vice). We did not propose hypotheses for the effects of terrorism on the salience of phrases corresponding to each of these foundations because there was insufficient theory and prior research to do so. The information from the CausalImpact analyses for these moral foundations can be found in Table 3; graphs for the salience trajectories for phrases corresponding to each of these foundations as a function of terrorism can be found in the supplemental materials.

## Discussion

To reiterate, the goal of this study was to investigate possible relations between the 9/11 terrorism attacks and moral language in books published in the United States, Great Britain, and in Spanish-speaking countries. Four hypotheses were tested concerning authority and loyalty phrases in books published in the United States. We predicted that authority- and loyalty-virtue phrases would be depressed by the 9/11 terrorist attacks while the salience of authority-vice and loyalty-vice phrases would be increased in books published in the United States. Of the four hypotheses, only one–concerning authority-vice phrases–failed to receive support from graphical inspection of trends and the interrupted time-series analyses.

The pattern observed in the United States is most like that found in books published in Great Britain; in both corpora, authority-virtue and loyalty-virtue phrases were lower than predicted from synthetic control models for years following the 9/11 terrorist attacks, with the salience of loyalty-vice increasing. Although there is evidence (both in Table 3 and in the Figures) that the salience of phrases related to authority and loyalty in books published in Spanish were affected by the 9/11 terrorist attacks, the effect seemed to be reflected in increases in authority-vice, authority-virtue, loyalty-vice, and loyalty-virtue. In supplementary analyses, following the Pearl Harbor attack, the salience of authority-vice and loyalty-vice increased in books published in the U.S. Authority-vice and loyalty-vice also increased in books published in Spanish following a terrorist bombing. Finally, supplementary analyses of the fiction corpus found that the 9/11 attack was associated with increases in authority-vice and loyalty-vice and decreases in authority-virtue. Across four corpora (books published in the U.S., books published in Great Britain, books published in Spanish, fiction) and three time periods (years surrounding Pearl Harbor, the Hipercor bombing in Spain, the 9/11 terrorist attacks), the moral salience of theory-relevant values changed. In all six analyses, the salience of loyalty-vice words increased following unexpected violent attacks.

Consistent with the interpretation that the 9/11 terrorist attacks contributed to the changes in the salience of moral foundation phrases in published books are the findings concerning changes in the salience of the word “terrorism” and in the salience of fear words in years after 2001. In years immediately following the 9/11 terrorist attacks, the relative frequency of “terrorism” in all three corpora increased dramatically. This suggests that terrorism was very much on the minds of the authors of books in the United States, Great Britain, and in Spanish-speaking countries. Similarly, the salience of fear words also increased substantially in all three corpora in years following the 9/11 attacks. Theories connecting terrorism to moral values and political beliefs often posit that fear and the perception of threat mediates the relation; as the fear of terrorism increases, the desire grows for strong leaders to defend the country from threats as does the distrust of those from other groups. The increase of fear-related phrases observed in this study in years following 2001 is consistent with these theoretical models.

### Limitations

One obstacle to causal inferences concerning terrorism and moral language in published books is that the time dimension is molar. Books often take years to write and publish, with this period varying substantially. Consequently, there is likely to be a lag of years between a terrorism event and the maximum impact of that event on word use in published books. Moreover, the statistical method used in this study (interrupted time-series implemented in the CausalImpact program) requires multiple time points before and after an event to detect reliable deflections in trajectories. Because the time points are measured in years in this study, the consequence is that the comparisons are of blocks of years to each other, not the days immediately preceding and following the 9/11 attacks. Because the nature of the data and the analysis techniques make it difficult to specify when the effects should be apparent and are initially discernible, the temporal linking of cause and effect is uncertain.

In future work the sensitivity of measures of moral language possibly could be improved through several different processes. For example, lemmatizing words and phrases in the moral dictionaries, and adding close alternatives to phrases, might allow moral meaning in texts to be better assessed in texts. These expanded dictionaries could then be validated against the dictionaries used in this study. An alternative and perhaps complementary strategy might be to train artificial intelligence (AI) tools with a subset of the ngrams identified in this study as containing moral content, and then use artificial intelligence tools to code new sets of data. This approach seems quite promising in small samples of data [32].

It’s also possible that the changes in the salience of phrases related to morality observed in this paper reflect broad transformations in the corpora. For example, Bollen and colleagues [29] have recently reported that the English-, Spanish-, and German-language corpora assembled by Google all are characterized by a dramatic increase in phrases suggestive of cognitive distortions (e.g., “everyone thinks I’m a loser”). The patterns in this research suggest dramatic increases over the years between 2000 and 2019 (nearly 2 SD units). In contrast, the trends observed in the study reported here suggest short-term deflections from long historical trends (Figs 3 and 4). Moreover, as noted earlier, our trends are consistent with those found in the corpus of fiction words, which is generally considered relatively robust for inferences of historical trends. Nonetheless, a great deal of work remains to be done in connecting word use in books to psychological functioning in people [33].

### Future directions

As noted earlier, the language in books both reflects contemporary concerns and is intended to influence readers. One direction for future work is to examine the relations between reading and the salience in the reader’s mind of different moral foundations. For example, it appears that the salience of authority-virtue phrases was much lower in books published in the United States in the 1970s than it is in books published in the 21st century. Are readers of 21st century books made more sensitive to authority moral values than readers of 1970s books by the more common mention of phrases related to the moral values? Alternatively, are books written in the 21st century more attractive to readers seeking moral messages?

It would also be interesting to examine the correspondence of deflections in the trajectories of phrase salience with cultural trends. An example of the direction that this kind of work might take is presented in the supplementary materials, which depicts the results of an analysis of moral foundation vice and virtue words in U.S. presidential inauguration addresses as a function of year. The graph in S3 Fig suggests that the salience of vice and virtue words in inaugural addresses follows trajectories like those identified in this paper and depicted in Fig 2. In future work, it might be interesting to investigate the effectiveness of presidential campaign speeches as a function of the relative salience of the moral foundations as characterized in this study. For example, Donald Trump’s depiction of moral chaos might have resonated less with voters less in 2000 than it did in 2016 when authority-vice phrases had become much more common in American books.

As an understanding of the connections of the contents of published books to psychological and cultural functioning grows, new research possibilities emerge for historical topics and areas of contemporary interest that escape more traditional methods such as surveys.

Files used to analyze data are available at: https://osf.io/brzjw/, DOI 10.17605/OSF.IO/BRZJW.

## Supporting information

S1 Table Observed values and synthetic control estimates, with confidence intervals, for deflections in moral foundation trajectories following 9/11 in works of fiction. (DOCX)

S2 Table Observed values and synthetic control estimates, with confidence intervals, for deflections in moral foundation trajectories following Pearl Harbor attack, 1941. (DOCX)

S3 Table Observed values and synthetic control estimates, with confidence intervals, for deflections in moral foundation trajectories following Hipercor bombing in Spain, 1987. (DOCX)

S1 Fig Salience of virtue phrases by year in fiction published in English. (TIF)

S2 Fig Salience of vice phrases by year in fiction published in English. (TIF)

S3 Fig Salience of vice and virtue by year in U.S. Presidential inaugural speeches.
(TIF)

S4 Fig Salience for care-virtue and care-vice phrases by country. (TIF)

S5 Fig Salience for fairness-virtue and fairness-vice phrases by country. (TIF)

S6 Fig Salience for sanctity-virtue and sanctity-vice phrases by country. (TIF)
