# Supplementary material for: The impact of terrorist attacks on cultural values as expressed in books
Source: PLoS One. 2024 Nov 22;19(11):e0311095. doi: 10.1371/journal.pone.0311095 (PMC11584079; doi:10.1371/journal.pone.0311095)
Supplement: S1 Table — (DOCX) [file pone.0311095.s001.docx]

**S3 Table. Observed Values and Synthetic Control Estimates, with Confidence Intervals, for Deflections in Moral Foundation Trajectories following 9/11 in Works of Fiction.**

|  |  |  | **Estimated Effect** |  |  |  |
| --- | --- | --- | --- | --- | --- | --- |
| **Foundation** | **Actual** | **Estimate** | **Causal**  **Effect** | **Lower Bound** | **Upper Bound** | **p** |
| Authority-Vice | 1.39 | 1.05 | 0.34 | 0.81 | 1.28 | 0.00 |
| Authority-Virtue | 1.19 | 1.50 | −0.30 | 1.32 | 1.68 | 0.00 |
| Loyalty-Vice | 1.94 | 1.02 | 0.92 | 0.75 | 1.30 | 0.00 |
| Loyalty-Virtue | 1.30 | 1.40 | −0.11 | 1.22 | 1.59 | 0.13 |
