# Supplementary material for: The impact of terrorist attacks on cultural values as expressed in books
Source: PLoS One. 2024 Nov 22;19(11):e0311095. doi: 10.1371/journal.pone.0311095 (PMC11584079; doi:10.1371/journal.pone.0311095)
Supplement: S2 Table — (DOCX) [file pone.0311095.s002.docx]

**S4 Table. Observed Values and Synthetic Control Estimates, with Confidence Intervals, for Deflections in Moral Foundation Trajectories following Pearl Harbor Attack, 1941.**

|  |  |  | **Estimated Effect** |  |  |  |
| --- | --- | --- | --- | --- | --- | --- |
| **Foundation** | **Actual** | **Estimate** | **Causal**  **Effect** | **Lower Bound** | **Upper Bound** | **p** |
| Authority-Vice | −0.05 | −0.27 | 0.22 | −0.50 | −0.03 | 0.04 |
| Authority-Virtue | 0.07 | 0.00 | 0.07 | −0.14 | 0.16 | 0.17 |
| Loyalty-Vice | −0.09 | −0.39 | 0.31 | −0.67 | −0.11 | 0.02 |
| Loyalty-Virtue | 0.13 | 0.12 | 0.00 | −0.07 | 0.33 | 0.49 |
