# Supplementary material for: The impact of terrorist attacks on cultural values as expressed in books
Source: PLoS One. 2024 Nov 22;19(11):e0311095. doi: 10.1371/journal.pone.0311095 (PMC11584079; doi:10.1371/journal.pone.0311095)
Supplement: S3 Table — (DOCX) [file pone.0311095.s003.docx]

**S5 Table. Observed Values and Synthetic Control Estimates, with Confidence Intervals, for Deflections in Moral Foundation Trajectories following Hipercor Bombing in Spain, 1987.**

|  |  |  | **Estimated Effect** |  |  |  |
| --- | --- | --- | --- | --- | --- | --- |
| **Foundation** | **Actual** | **Estimate** | **Causal**  **Effect** | **Lower Bound** | **Upper Bound** | **p** |
| Authority-Vice | 1.11 | 1.80 | −0.69 | 1.44 | 2.19 | 0.00 |
| Authority-Virtue | 0.37 | −0.01 | 0.37 | −0.21 | 0.22 | 0.00 |
| Loyalty-Vice | 0.95 | 1.52 | −0.58 | 1.11 | 1.99 | 0.01 |
| Loyalty-Virtue | 0.66 | −0.14 | 0.80 | −0.55 | 0.32 | 0.00 |
