# Supplementary figures and images for: The impact of terrorist attacks on cultural values as expressed in books
Source: PLoS One. 2024 Nov 22;19(11):e0311095. doi: 10.1371/journal.pone.0311095 (PMC11584079; doi:10.1371/journal.pone.0311095)

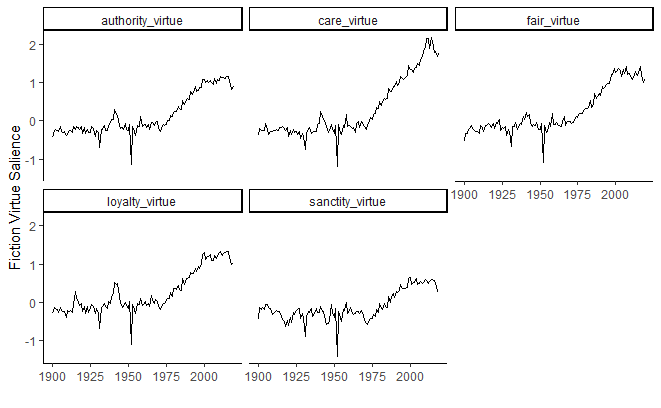

Supplement: S1 Fig — (TIF) [file pone.0311095.s004.tif]

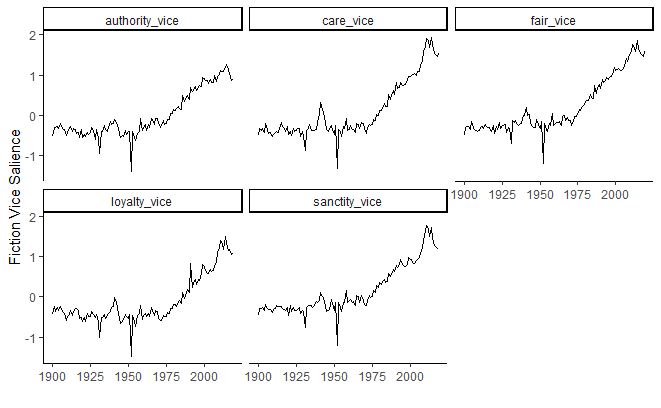

Supplement: S2 Fig — (TIF) [file pone.0311095.s005.tif]

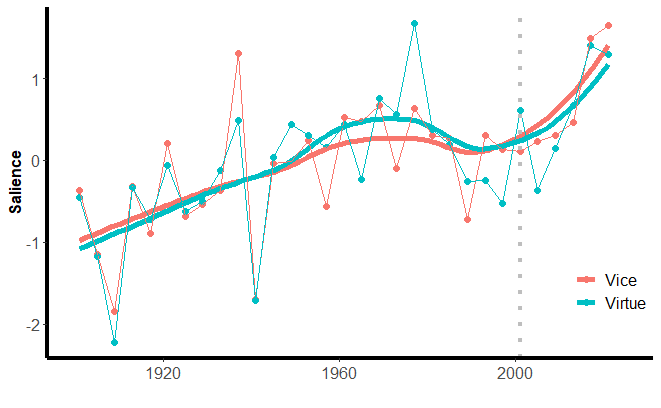

Supplement: S3 Fig — Presidential inaugural speeches. (TIF) [file pone.0311095.s006.tif]

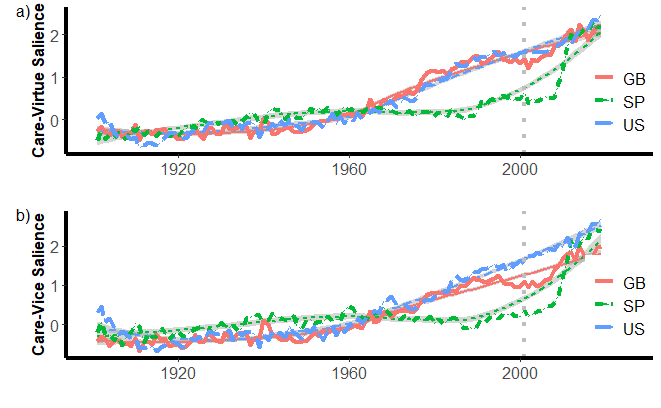

Supplement: S4 Fig — (TIF) [file pone.0311095.s007.tif]

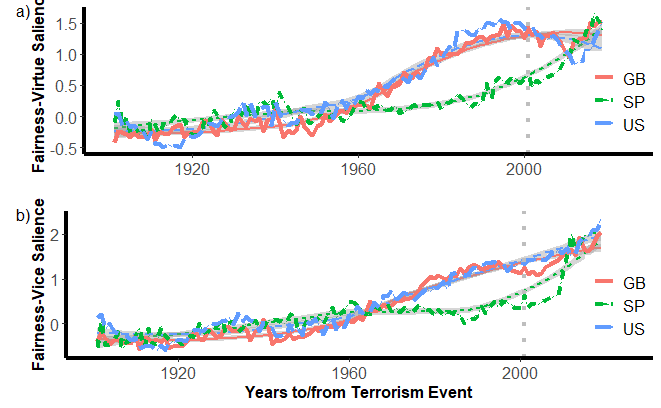

Supplement: S5 Fig — (TIF) [file pone.0311095.s008.tif]

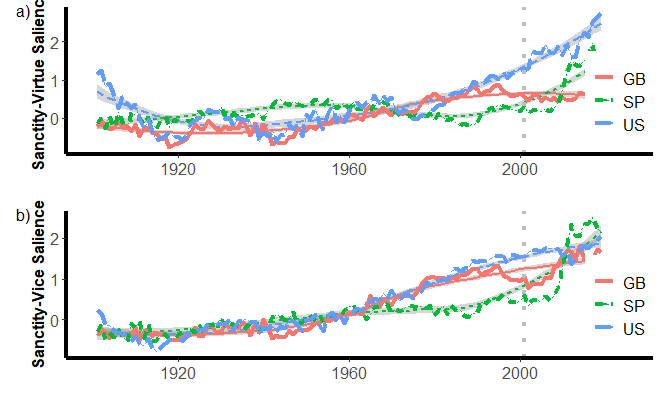

Supplement: S6 Fig — (TIF) [file pone.0311095.s009.tif]
